# Supplementary material for: Prognostic Significance of Glucose Metabolism as GLUT1 in Patients with Pulmonary Pleomorphic Carcinoma
Source: J Clin Med. 2020 Feb 3;9(2):413. doi: 10.3390/jcm9020413 (PMC7074371; doi:10.3390/jcm9020413)

Figure S1. Each percentages of epithelial and sarcomatous components

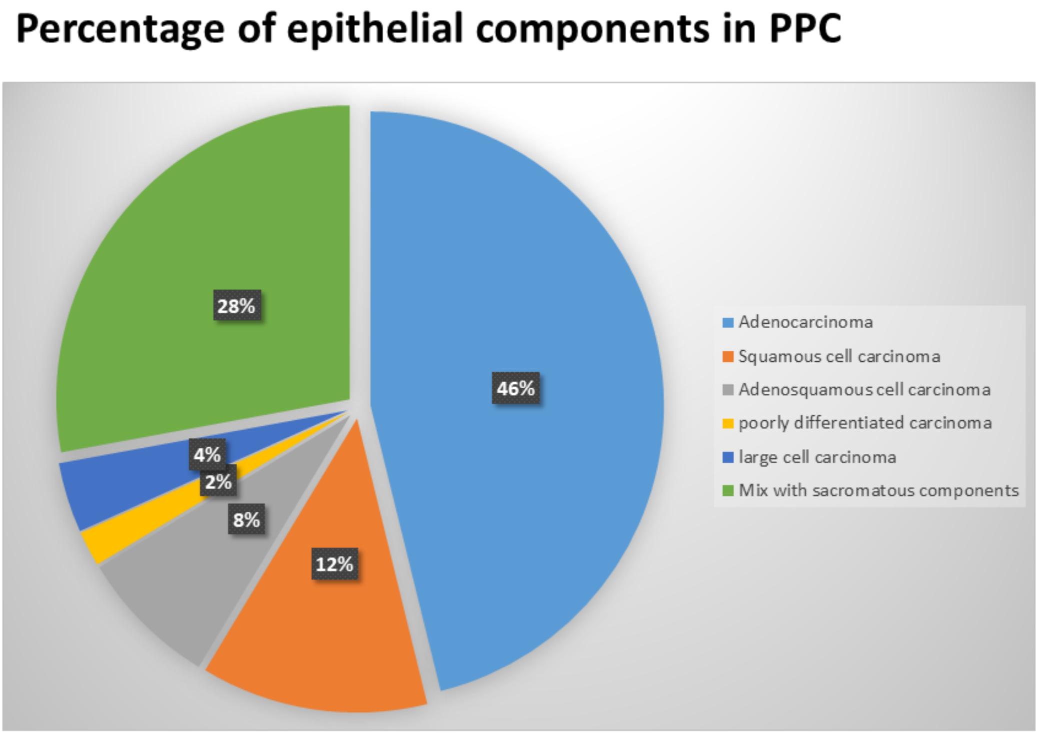

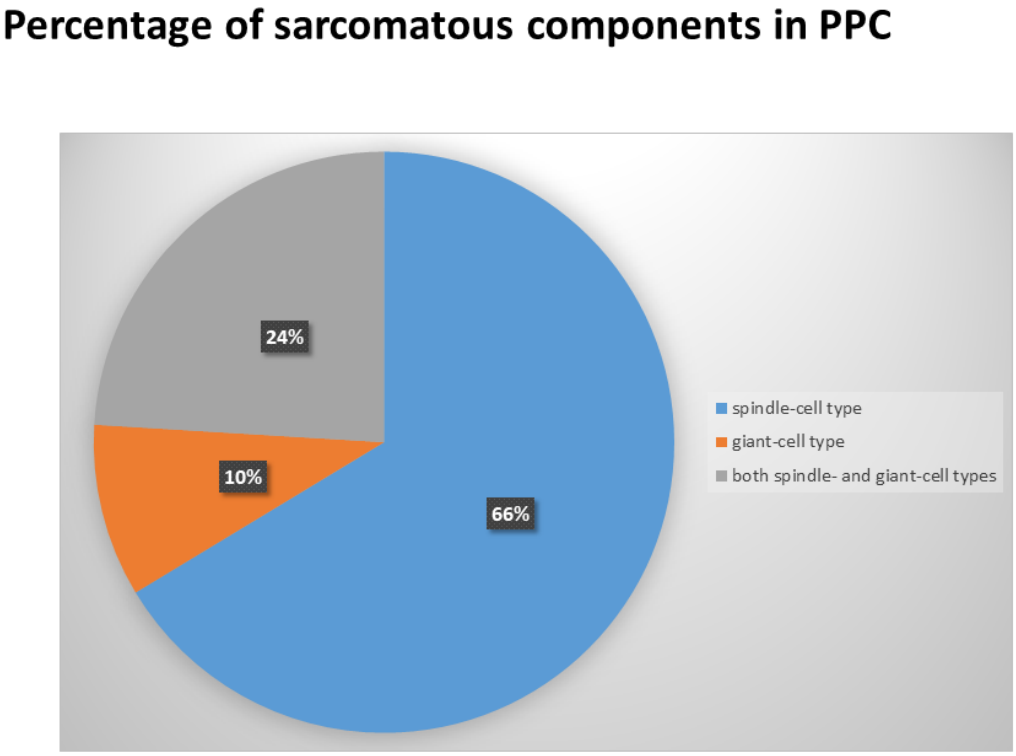

Supplement: Supplementary file 1 [file jcm-09-00413-s001.pdf]
